# Influence of metal-mediated aerosol-phase oxidation on secondary organic aerosol formation from the ozonolysis and OH-oxidation of α-pinene

**DOI:** 10.1038/srep40311

**Published:** 2017-01-06

**Authors:** Biwu Chu, John Liggio, Yongchun Liu, Hong He, Hideto Takekawa, Shao-Meng Li, Jiming Hao

**Affiliations:** 1 State Key Joint Laboratory of Environment Simulation and Pollution Control, Research Center for Eco-Environmental Sciences, Chinese Academy of Sciences, Beijing 100085, China; 2 Center for Excellence in Regional Atmospheric Environment, Institute of Urban Environment, Chinese Academy of Sciences, Xiamen 361021, China; 3 Air Quality Research Division, Environment Canada, Toronto, Ontario M3H5T4, Canada; 4 University of Chinese Academy of Sciences, Beijing 100049, China; 5 Toyota Central Research and Development Laboratory, Nagakute, Aichi 480-1192, Japan; 6 State Key Joint Laboratory of Environment Simulation and Pollution Control, School of Environment, Tsinghua University, Beijing 100084, China

## Abstract

The organic component is the most abundant fraction of atmospheric submicron particles, while the formation mechanisms of secondary organic aerosol (SOA) are not fully understood. The effects of sulfate seed aerosols on SOA formation were investigated with a series of experiments carried out using a 9 m^3^ smog chamber. The presence of FeSO_4_ or Fe_2_(SO_4_)_3_ seed aerosols decreased SOA yields and increased oxidation levels in both ozonolysis and OH-oxidation of α-pinene compared to that in the presence of ZnSO_4_ or (NH_4_)_2_SO_4_. These findings were explained by metal-mediated aerosol-phase oxidation of organics: reactive radicals were generated on FeSO_4_ or Fe_2_(SO_4_)_3_ seed aerosols and reacted further with the organic mass. This effect would help to explain the high O/C ratios of organics in ambient particles that thus far cannot be reproduced in laboratory and model studies. In addition, the gap in the SOA yields between experiments with different seed aerosols was more significant in OH-oxidation experiments compared to ozonolysis experiments, while the gap in estimated O/C ratios was less obvious. This may have resulted from the different chemical compositions and oxidation levels of the SOA generated in the two systems, which affect the branching ratio of functionalization and fragmentation during aerosol oxidation.

Atmospheric fine particles can affect human health, atmospheric visibility, and the global radiative balance^1^,^2^,^3^,^4^,^5^,^6^. The organic component is the most abundant fraction of atmospheric submicron particles on a regional and global basis^7^,^8^. It is estimated that more than half of the total organic carbon in aerosol is secondary in nature^7^,^9^. However, the formation mechanisms of secondary organic aerosol (SOA) in the atmosphere are not fully understood, resulting in difficulties in accurately simulating SOA in air quality models^10^,^11^.

SOA yields from volatile organic compounds (VOCs) depend upon atmospheric conditions. A number of factors, including temperature^12^, RH^13^, the ratio of hydrocarbons to NO_*x*_^14^, and the presence of SO_2_ or NH_3_^15^,^16^,^17^ have important influences on SOA formation. In addition, the presence of pre-existing aerosols also plays an important role in both heterogeneous and aerosol-phase reactions, by shifting gas-particle partitioning and thus influencing overall SOA yields. Acidic inorganic seed aerosols have been found to enhance SOA mass during the oxidation of VOCs through acid-catalyzed aerosol-phase reactions^18^,^19^, resulting in the production of products with low volatility, including oligomers^20^,^21^,^22^. Recently, metallic sulfates and mineral dust aerosols have also been found to either suppress or enhance SOA formation^23^,^24^,^25^. These studies implied that the effects of seed aerosols on SOA formation must be considered in order to understand SOA generation mechanisms in the atmosphere.

In addition to the uncertainties associated with SOA formation mechanisms, the aging and oxidation of SOA formed in aerosols is poorly understood. The high oxidation level of organics in ambient particles, typically characterized by the O/C ratio, thus far cannot be reproduced in model and laboratory studies^7^,^26^. As a result of the complexity of oxidation chemistry in multiple phases, the oxidation state of SOA is a key property, as it can be related to the volatility^27^, hygroscopicity^28^,^29^, density^30^, and optical properties^31^ of the various components in the aerosols. The oxidation level is also a key property in differentiating primary organic aerosol (POA) from SOA in aerosol mass spectrometry (AMS) measurements^7^,^26^. The competition between functionalization and fragmentation was found to be highly related to the oxidation state of SOA^30^,^32^,^33^. Functionalization usually decreases the volatility of organics, while fragmentation leads to formation of products with increased saturation vapor pressures (thus higher volatilities). Therefore, the oxidation state of SOA can influence the mass loading of SOA in the atmosphere, and is an important parameter required to evaluate the fate of SOA as a result of atmospheric aging. While the oxidation state of SOA is mainly dependent upon the concentration and type of the oxidant and the aging time, it may be also influenced by other factors such as seed aerosol composition^34^,^35^.

High concentrations of metallic species are often observed in atmospheric aerosols in polluted cities. Zinc and iron are known to be the most abundant transition metals in the atmosphere^36^,^37^,^38^. The average zinc concentration has been reported to range from dozens to hundreds of ng m^−3^ in the atmosphere^36^,^38^,^39^,^40^,^41^, and was found to be associated with submicron particles^42^ in sulfate and nitrate forms^43^,^44^. These metal-containing aerosols may interact with photochemical precursors and subsequently influence the SOA formation^24^. Iron exists in both Fe(II) and Fe(III) chemical forms under specific atmospheric conditions^45^. Due to its different oxidation states, iron is believed to participate in atmospheric processes in aerosols that alter aerosol composition^46^. For example, iron catalyzes SO_2_ oxidation in the atmosphere^47^,^48^, a pathway that contributes a considerable proportion to the global atmospheric sulfate burden^49^. In addition, particulate iron has important catalytic effects on reactions through Fenton-like chemistry that produces reactive oxygen species (ROS), including hydroxyl radicals and hydrogen peroxide in aerosols^35^,^50^,^51^,^52^. These ROS may influence SOA formation through reactions with condensed organic mass^35^,^53^. Recently, a few studies demonstrated that gaseous compounds could be taken up and oxidized on the aerosol liquid water interfaces^54^,^55^, and the Fenton chemistry was found to be faster at interfaces than in the bulk^56^. Several studies have documented the photo-reduction of Fe(III) to Fe(II) in the presence of organic compounds in clouds and fog^46^,^57^,^58^, and it is likely that such reduction also occurs in dark reactions^49^.

In previous studies we have demonstrated that SOA mass from α-pinene photooxidation decreased in the presence of FeSO_4_ seed aerosols compared to experiments that were seed-free or with (NH_4_)_2_SO_4_ seed aerosols^25^,^59^. Due to the limitations of analytical instruments, the concentrations of precursors of SOA were very high (ppm level) in these studies. Some key aspects, for example, the O/C ratio of SOA, the experimental error, the comparison between effects of Fe(II) and Fe(III), and the comparison between photooxidation and dark experiments, were not investigated. The reducing nature of Fe(II) had been proposed to explain the suppressing effect of FeSO_4_ seed aerosols on SOA formation in previous studies, but it seems an unlikely explanation according to the experimental results in this study. In the current work, our objective is to further investigate the effect of metal-mediated aerosol-phase oxidation on the yield and oxidation state of SOA from both ozonolysis and OH-oxidation under normal environmental concentrations. The change in SOA yield and oxidation level of organics in experiments with metallic sulfate seed aerosols, including FeSO_4_, Fe_2_(SO_4_)_3_ and ZnSO_4_, compared to that with nonmetallic seed aerosols, are reported and quantitatively compared between ozonolysis experiments and OH-oxidation experiments.

## Results

### SOA formation from ozonolysis of α-pinene

A series of α-pinene ozonolysis experiments were carried out under similar conditions in the presence of sulfate seed aerosols close to normal environmental concentrations. The experimental details are introduced in Table S1 in Supplementary Information. After 4.5 hours of reaction, approximately 4.7 ppb of α-pinene was consumed consistently in the experiments. No scavenger for free radicals was used in these experiments. According to the time variations of ozone and α-pinene, as well as their reaction rate constant, it was estimated that about 50–60% of the consumed α-pinene reacted with ozone. The concentrations or reagent ion normalized signals (which are proportional to concentrations) for α-pinene, acetone, pinonaldehyde and pinic acid (using the m/z 71 fragment^60^) and many other gas product fragments were not significantly different between experiments utilizing different types of sulfate seed aerosols. The differences between the average relative abundance of these gaseous products in experiments with different types of seed aerosol were less than 10%, with some examples shown in Figure S2 in the supplementary information. This suggests that the effect of seed aerosol type on gas phase reactions was not significant enough to be detected by the proton transfer reaction mass spectrometer (PTR-MS), and that the precursor concentration, oxidant levels and gas-phase wall losses between experiments were reproducible.

**Figure 1:**
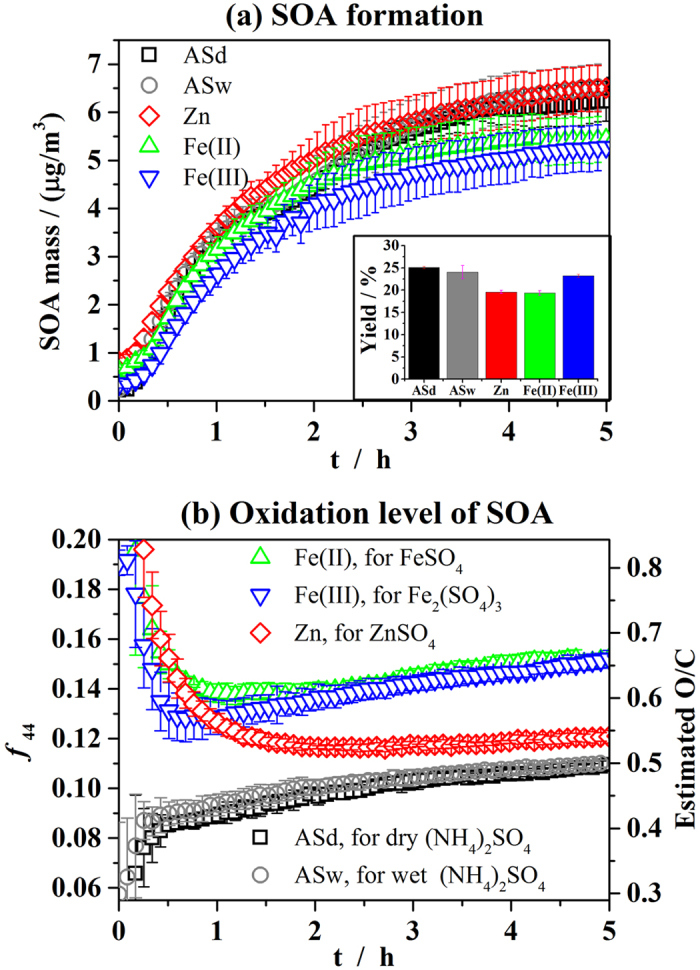
SOA growth (**a**), SOA yields (inset of (**a**)), and oxidation level of SOA (**b**) in ozonolysis of α-pinene with different sulfate seed aerosols. The error bar in the figure is one standard deviation based on three repeated experiments.

The organic mass concentration on the aerosols measured by aerosol mass spectrometer (AMS), due entirely to SOA formation, is plotted as a function of the reaction time in Fig. 1(a). The error bar in the figure is one standard deviation based on three repeated experiments. In this study, the wall losses of the aerosols were corrected using sulfate as a tracer for the AMS data^61^, while the data measured by the scan mobility particle sizer (SMPS) were corrected using the measured deposition rate in our previous study^59^, following the method developed by Takekawa *et al*.^12^. As shown in Fig. 1(a), the presence of dry (NH_4_)_2_SO_4_, wet (NH_4_)_2_SO_4_ or ZnSO_4_ seed aerosols resulted in slightly higher SOA formation than for FeSO_4_ or Fe_2_(SO_4_)_3_ seed aerosols. However, these differences were found to be not significant according to the statistical analysis. The reason for this is that the initial α-pinene and ozone concentrations for the repeated experiments were not identical, resulting in non-identical α-pinene consumption and high relative standard errors (RSEs) for SOA mass. As shown in the inset pictures of Fig. 1(a), the RSEs of the SOA yields (the mass ratio of the generated SOA and consumed α-pinene) were found to be much lower than those for SOA mass. SOA yields in experiments in the presence of FeSO_4_ or Fe_2_(SO_4_)_3_ seed aerosols were about 20% lower than those for (NH_4_)_2_SO_4_ or ZnSO_4_ seed aerosols. These differences were statistically significant according to one-way ANOVA statistical analysis results and means comparison with the Dunn-Sidak test, the details of which can be found in the Supplementary Information. Assuming a density of 1.4 g cm^−3^ for SOA^62^, SMPS gave similar SOA mass concentrations to AMS. The variations in precursor concentrations and oxidant levels among experiments with different types of sulfate seed aerosols were on average less than 5%. The FeSO_4_ or Fe_2_(SO_4_)_3_ seed aerosols likely accounted for the lower SOA yields in these experiments.

### Oxidation level of SOA from the ozonolysis of α-pinene

The cause of the varying SOA yields in the presence of different sulfate seed aerosol types was examined through the analysis of individual organic aerosol mass spectra. The fragment with mass to charge ratio of 44 (m/z 44), arising mostly from CO_2_^+^, has been attributed to highly oxidized organic components^63^, such as carboxylic acids and acyl peroxides, and its ratio to the total organic signal (*f*_44_) is often used to infer the degree of oxidation of SOA^26^,^64^. In this study, the O/C atomic ratio was estimated using the *f*_44_ and the correlation derived by Aiken *et al*.^65^.

Despite small SOA mass differences between experiments, very different profiles for the *f*_44_ and estimated O/C ratios were observed when conducting experiments with differing sulfate seed aerosols. In the first half hour of the experiment, the *f*_44_ and estimated O/C ratios were highly uncertain, since organic aerosol concentration in this period is very low. The seed aerosols rather than the generated SOA had a large contribution to the *f*_44_ and estimated O/C ratios in this period. The *f*_44_ values for metal solutions and ammonium sulfate solutions are shown in Figure S5 in the Supplementary Information. As the reaction continued, the effect from seed aerosols became insignificant since the amount of generated SOA was enormous compared to the organics introduced together with seed aerosols. In the presence of (NH_4_)_2_SO_4_, the oxidation level of SOA (as demonstrated by *f*_44_ and O/C) consistently increased. Conversely, the oxidation level of organics decreased dramatically in the first few minutes on ZnSO_4_, FeSO_4_ and Fe_2_(SO_4_)_3_ particles. As the reaction continued, the organic oxidation level on the ZnSO_4_ seed aerosols decreased further, becoming similar to that of the organics on the (NH_4_)_2_SO_4_ seed aerosols. The oxidation level of organics on the FeSO_4_ and Fe_2_(SO_4_)_3_ seed aerosols remained higher than that on the (NH_4_)_2_SO_4_ seed aerosols during the whole reaction.

To explain the higher oxidation levels of organics on the FeSO_4_ and Fe_2_(SO_4_)_3_ seed aerosols relative to the other seed aerosols, the condensed phase oxidation of organics is hypothesized to be responsible. Oxidation of condensed phase organics by gas phase radicals may also be possible and is summarized by George and Abbatt^66^. However, it is unlikely to result in significant differences for the organics partitioned to the different seed aerosols in this case because OH radical uptake on inorganics is relatively inefficient if the surface cannot be oxidized^66^. In addition, the organics generated in the gas phase should be similar among the experiments with different seed aerosols, and are unlikely to cause significant differences in the uptake of OH radical. It is further hypothesized that the iron cations in the seed aerosols are involved in the condensed phase oxidation. At 50% RH, FeSO_4_ and Fe_2_(SO_4_)_3_ aerosols both demonstrated some hygroscopic growth, as shown in Figure S1 in Supplementary Information. The aerosols were in a metastable state between the crystalline and liquid phase. Consequently, there were likely aqueous-phase layers on the surface of these two seed aerosols during these experiments. Deguillaume *et al*.^67^ provided a thorough review of the possible aqueous phase chemistry of Fe^2+^ and Fe^3+^ involving radicals and peroxides in the aqueous phase. Free radicals, including OH, can be formed from catalytic cycling of Fe^2+^ and Fe^3+^ in the aqueous phase of the ferric and ferrous sulfate aerosols. These radicals can react with the organic mass from the partitioning of gas-phase products of α-pinene oxidation on the seed aerosols, producing molecules that may contain more oxygen atoms than the original compounds. Further oxidation of SOA may also lead to some fragmentation of the condensed organics, which in the extreme case release more volatile organic compounds or even CO_2_ to the gas phase. These fragmentation reactions should be responsible for the lower SOA yields in experiments in the presence of FeSO_4_ and Fe_2_(SO_4_)_3_ seed aerosols relative to the other seed aerosols. Release of CO_2_ to the gas phase might be an important carbon loss path from the aerosol phase, since the differences in gaseous products in experiments with different types of seed aerosol were not significant.

### SOA formation from OH-oxidation of α-pinene

**Figure 2:**
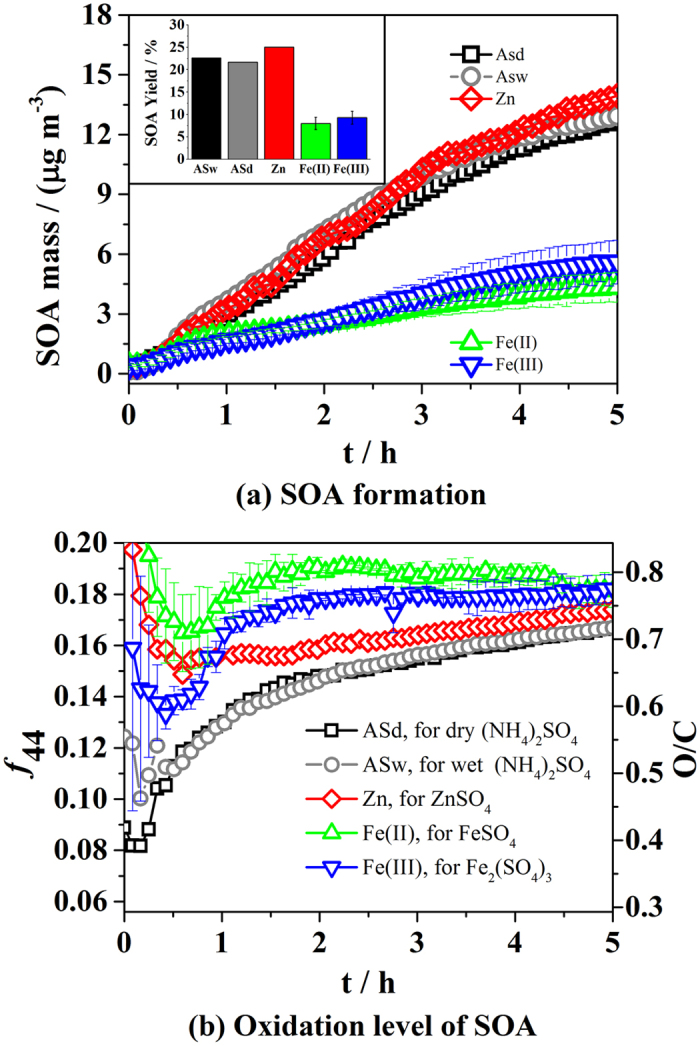
SOA growth (**a**), SOA yields (inset of (**a**)), and oxidation level of SOA (**b**) in the OH-oxidation of α-pinene with different sulfate seed aerosols. The error bar in the figure is one standard deviation based on three repeated experiments.

The effects of FeSO_4_ and Fe_2_(SO_4_)_3_ seed aerosols on SOA formation were further investigated in α-pinene OH-oxidation experiments. A series of experiments were carried out under similar conditions but with different seed aerosols, i.e. (NH_4_)_2_SO_4_, ZnSO_4_, FeSO_4_ or Fe_2_(SO_4_)_3_, and the experimental details are listed in Table S2 in Supplementary Information. Similar to what was observed in the ozonolysis experiments, the time variation for gas phase precursors and products was similar between experiments, as demonstrated in Figure S3 in Supplementary Information. The average relative abundances of the gaseous products (inset of Figure S3) in experiments in the presence of FeSO_4_ or Fe_2_(SO_4_)_3_ seed aerosols were similar to other experiments, with relative differences less than 15%. However, the SOA production varied significantly in the photooxidation experiments with different types of sulfate seed aerosols, as shown in Fig. 2(a). Similarly, the error bar in this figure is one standard deviation based on three repeated experiments. In experiments with FeSO_4_ or Fe_2_(SO_4_)_3_ seed aerosols, the growth of organic aerosols was significantly slower than that in experiments with (NH_4_)_2_SO_4_ or ZnSO_4_ seed aerosols. As a result, after 5 hours of reaction, the SOA concentrations in experiments with (NH_4_)_2_SO_4_ or ZnSO_4_ seed aerosols were at least two times higher than those in experiments with the FeSO_4_ or Fe_2_(SO_4_)_3_ seed aerosols. Since similar amounts of α-pinene were consumed in these experiments, SOA yields decreased by about 60% in experiments with the FeSO_4_ or Fe_2_(SO_4_)_3_ seed aerosols compared to the seed-free experiment or experiments with (NH_4_)_2_SO_4_ or ZnSO_4_ seed aerosols, which was much higher than the decrease in yield (about 20%) observed in ozone experiments.

### Oxidation level of SOA from the OH-oxidation of α-pinene

The ratio of m/z 44 to the total organic signal (*f*_44_) and O/C atomic ratio as a function of reaction time are shown in Fig. 2(b). In the experiments with (NH_4_)_2_SO_4_ seed aerosols, there was a significant increase in *f*_44_ over time, consistent with numerous reports of SOA formation in chamber and field studies^26^,^30^,^65^. In contrast, SOA generated in experiments with the FeSO_4_ or Fe_2_(SO_4_)_3_ seed aerosols were significantly more oxidized than that in experiments with the (NH_4_)_2_SO_4_ seed aerosols. It is interesting that the degree of SOA oxidation in FeSO_4_ or Fe_2_(SO_4_)_3_ experiments increased in a short time, with the *f*_44_ increasing to a plateau in about 45 minutes, as shown in both Figs 2 and S4. Such a rapid oxidation may be caused by oxidation with a high concentration of ROS in the condensed phase on the FeSO_4_ or Fe_2_(SO_4_)_3_ seed aerosols. In addition to the Fenton reaction^68^, photolysis of Fe^3+^ complexes^69^ in the aqueous phase can also produce OH radicals under irradiation. As described by Deguillaume *et al*.^67^ and references therein, the relative importance of Fenton reactions and the photolysis of Fe^3+^ complexes in the production of OH radicals in solution remains unclear but is likely dependent upon many factors, including iron concentration and pH. In this study, the high concentration of iron ion may provide favorable conditions for the generation of free radicals. Besides, the plateau of *f*_44_ and O/C in the photooxidation experiments is different from the consistent increase in *f*_44_ and O/C over time in ozonolysis experiments in the presence of FeSO_4_ or Fe_2_(SO_4_)_3_ seed aerosols. The increase of *f*_44_ and O/C might be suppressed by the photolysis of Fe^3+^ complexes^53^, since release of CO_2_ to the gas phase might occur and decrease the *f*_44_ and O/C of the organic aerosol. The fragmentation of the condensed organics due to oxidation with ROS and the photolysis of Fe^3+^ complexes may both contribute to the reduced SOA yields in FeSO_4_ and Fe_2_(SO_4_)_3_ seed aerosol experiments.

### Overall effect of FeSO_4_ or Fe_2_(SO_4_)_3_ on SOA formation

**Figure 3:**
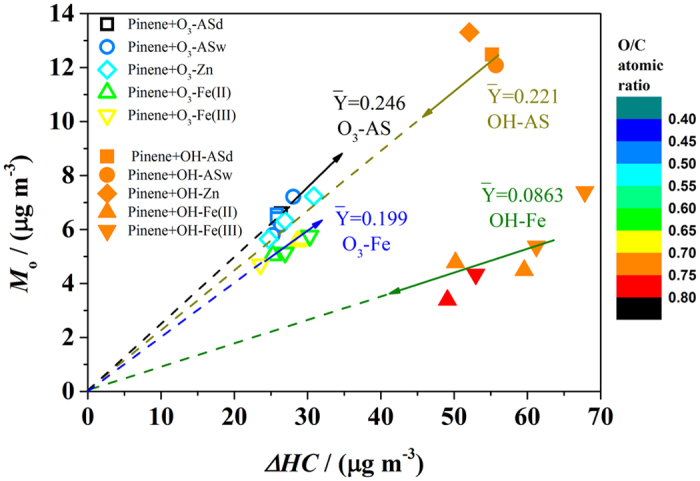
SOA generation (*M*_*o*_) as a function of reacted α-pinene (*ΔHC*) in ozonolysis and OH-oxidation of α-pinene with different sulfate seed aerosols. The slopes of the dashed lines indicate the average SOA yields. The color of the points indicates the O/C ratio of the SOA.

While a lower SOA yield was measured in experiments with the FeSO_4_ or Fe_2_(SO_4_)_3_ seed aerosols relative to that with (NH_4_)_2_SO_4_ for both the ozonolysis and the OH-oxidation of α-pinene, the extent of this effect (between O_3_ and OH experiments) was different. The average SOA yields (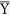) for each set of experiments are shown in Fig. 3. Since there was no pre-existing organic aerosol mass in any of the experiments, and the *M*_*o*_ were similar among each set of experiments with similar experimental conditions, the dependence of SOA yield upon the organic aerosol mass was not taken into account. In the ozonolysis of α-pinene, the SOA yield decreased from 0.246 ± 0.011 in experiments using the (NH_4_)_2_SO_4_ seed aerosols to 0.199 ± 0.014 in experiments with the FeSO_4_ or Fe_2_(SO_4_)_3_ seed aerosols, a decrease of 19%. The uncertainties of SOA yields were one standard deviation based on SOA yields from three repeated experiments. This is statistically significant at the 0.05 level as determined using an ANOVA statistical analysis. However, for the OH-oxidation of α-pinene, SOA yields decreased more significantly between experiments, from 0.221 to 0.086 ± 0.014; a decrease of around 61%. As we noted above, ozonolysis experiments may be influenced somewhat by OH radicals formed during the reaction. Therefore, the observed differences between the OH-oxidation experiments and the ozonolysis experiments in the effects of FeSO_4_ and Fe_2_(SO_4_)_3_ seed aerosols on SOA formation are likely a lower limit of the true differences. The presence of the FeSO_4_ or Fe_2_(SO_4_)_3_ seed aerosols also resulted in a statistically significant increase in oxidation level (relative to when the (NH_4_)_2_SO_4_ seed aerosols were used) in the ozonolysis of α-pinene, while this was not prominent in OH-oxidation experiments.

These differences in mass and SOA oxidation level may be explained by the competition between fragmentation and functionalization, whereby fragmentation and functionalization lead to a decrease of SOA mass and increase in oxidation level, respectively^30^,^32^,^33^. The branching ratio between fragmentation and functionalization is uncertain, but was found to increase as O/C rises^7^,^30^,^70^. The SOA was less oxidized in ozonolysis experiments than that in OH-oxidation experiments, as demonstrated in Figs 3 and S4. The lower oxidation level of the products might lead to a lower branching ratio between fragmentation and functionalization during the oxidation in the condensed phase with FeSO_4_ or Fe_2_(SO_4_)_3_ seed aerosols, resulting in more-oxidized SOA and slightly lower SOA yield in ozonolysis experiments with FeSO_4_ or Fe_2_(SO_4_)_3_ seed aerosols compared to ozonolysis experiments with (NH_4_)_2_SO_4_ seed aerosols. In contrast, the high branching ratio between fragmentation and functionalization resulted in much less SOA mass and a little higher O/C in OH-oxidation experiments with FeSO_4_ or Fe_2_(SO_4_)_3_ seed aerosols relative to that in OH-oxidation experiments with (NH_4_)_2_SO_4_ seed aerosols.

Another possible reason for the differences in the change of oxidation level and SOA yields between ozonolysis and OH-oxidation experiments is a different amount of OH radicals in the condensed phase. With abundant peroxides in the oxidation of α-pinene^71^,^72^ and similar conditions of seed aerosol and RH, OH radicals generated in the condensed phase were assumed to be similar between ozonolysis and OH-oxidation experiments. The differences caused by FeSO_4_ or Fe_2_(SO_4_)_3_ between ozonolysis experiments and OH-oxidation experiments were likely to be due to differences in the initial chemical composition of the condensed SOA before it was partially fragmented by OH radical in the aerosol phase. Recently, iron-carboxylate complex photolysis was reported to be an important sink for carboxylic acids^73^. Thus, the presence of light may also contribute to the decrease in SOA yield in the OH-oxidation experiments in the presence of FeSO_4_ or Fe_2_(SO_4_)_3_ seed aerosols with highly oxidized SOA^53^.

## Discussion

The results suggest that iron-containing sulfate seed aerosols, i.e. FeSO_4_ or Fe_2_(SO_4_)_3_, could have a substantial impact on the formation and properties of SOA. The effects of FeSO_4_ seed aerosol on SOA in OH-oxidation experiments were similar to those in previous studies^25^,^59^, but the decrease percentages of SOA mass were higher than in previous studies (60% vs. 8–34%) with similar concentrations of FeSO_4_ seed aerosols. A detailed comparison of this study with other studies is introduced in Table S6 in the Supplementary information. The higher percentage decrease in this study occurred because most of the SOA were generated on the surface of FeSO_4_ seed aerosols and were involved in the metal-mediated aerosol-phase oxidation in this study, while in previous studies the new particle formation was not controlled. What’s more, FeSO_4_ and Fe_2_(SO_4_)_3_ showed very similar effects on SOA mass and the O/C ratio in this study, indicating a cyclic oxidation-reduction of the iron. The generation of ROS in the aerosol phase can lead to rapid and efficient oxidation of SOA, resulting in oxidation level increase and aerosol mass loss. UV irradiation is not necessary for this effect since it was observed in both dark ozonolysis experiments and HONO photo-oxidation experiments. Most of the experiments were repeated three times to quantify the experimental errors, and this effect was found to be statistically significant. So this effect may need to be considered in simulations of SOA formation, and would help to explain the high O/C of organics in ambient particles that thus far have not been able to be reproduced in laboratory and model studies. In addition, these effects were found to be related to the initial properties of the SOA. The aerosol mass loss in the oxidation is more pronounced for highly oxidized SOA compared to SOA with lower oxidation level, due to the presence of aerosol-phase ROS. The concentrations of ROS within atmospheric particles and their effects on different families of SOA are critical for the improved characterization of aerosol-phase processes of atmospheric organic aerosol.

One thing we should point out is that the initial concentrations of reactants in this study were higher than those present in the atmosphere. This served to generate high enough SOA concentrations to reduce the experimental uncertainty. The organic aerosol mass loading was comparable to that in the ambient atmosphere, and only one hydrocarbon was used in the experiments. According to a comparison of this study with previous studies, with details in Table S6 in the Supplementary Information, the suppressing effect of FeSO_4_ on SOA mass was more significant with lower SOA mass loading. The concentrations of iron in the experiments were calculated and listed in Table S3 in the Supplementary Information. Although the total mass concentrations of iron in this study were lower than in our previous studies, and were comparable to that in the atmosphere in polluted cities, the water-soluble iron concentrations and the iron concentrations in the particle phase were higher than those under normal ambient conditions. The high water-soluble iron concentration is likely to cause an overestimation of the effect of iron sulfate on SOA formation, while the high iron concentration in the particle phase might cause an underestimation of the effect. This is discussed in detail in the Supplementary Information. The influence of metal-mediated aerosol-phase oxidation on secondary organic aerosol formation would be most significant with a low SOA loading and a high concentration of highly dispersed water-soluble iron.

## Methods

### Chamber facility

Experiments were carried out in a 9 m^3^ cylindrical reactor, constructed from Teflon film and irradiated by 24 Sylvania black-light lamps (365 nm). A detailed description of the chamber has been published elsewhere^74^. The chamber was connected to a proton transfer reaction mass spectrometer (PTR-MS, IONICON Analytik) and an O_3_ monitor (2B Technologies) to measure gas-phase organic compounds and ozone. A negative-ion proton-transfer chemical-ionization mass spectrometer (NI-PT-CIMS) was used to measure the initial concentration of HONO in each experiment to create similar gas-phase oxidation environments among the OH-oxidation experiments. The NI-PT-CIMS has been described in detail elsewhere^75^. Aerosols in the chamber were measured with a scan mobility particle sizer (SMPS, TSI Model 3080) and a compact time-of-flight aerosol mass spectrometer (AMS, Aerodyne Research, Inc. C-ToF-AMS)^76^ to obtain the information regarding the size distribution and chemical composition of the aerosols.

### Seed aerosol and oxidant generation

Sulfate seed aerosols were generated by atomizing 1 g L^−1^ of zinc sulfate, ferrous sulfate and ferric sulfate solutions using a constant output atomizer (TSI Model 3076). The generated droplets were passed through a diffusion dryer (TSI Model 3062), where the RH was below 10% at the exit, to obtain dry seed aerosols. A differential mobility analyzer (DMA, TSI Model 3081) was used to size select the seed aerosols (72 nm). Wet seed aerosols were obtained by directly introducing droplets into the DMA for size selection (79 nm). According to the SMPS measurements, most of the dry or wet seed aerosols (approximately 75% in number) had a diameter of 76 ± 5 nm at 50% RH in the chamber. O_3_ was generated from zero air in a UV ozone generator (OG-1, PCI Ozone Corp.). OH radical was generated from photolysis of HONO, while HONO was generated by passing a HCl-containing gas stream through a tube containing NaNO_2_ salt granules, as described by Roberts *et al*.^77^.

### Experimental procedure

In the ozonolysis experiments, the seed aerosols were introduced followed by O_3_. Gaseous α-pinene was generated by injection of liquid α-pinene into a vaporizer where a large flow of zero air carried the α-pinene vapor into the chamber to begin the reaction. In the OH-oxidation experiments, the seed aerosols were also introduced first, followed by HONO and α-pinene in a stream of zero air. Turning on the UV lights initiated the formation of OH radicals and was considered the reaction starting point. Due to the limited volume for sampling in the chamber and the ongoing particle deposition during the reaction, the experiments were stopped after approximately 5 hours of reaction. After 5 hours of α-pinene oxidation in both the ozonolysis and OH-oxidation experiments, a plateau in the concentration of organic aerosols was apparent. Due to the low concentrations of α-pinene and oxidants, as well as the sufficient surface area provided by the seed aerosols, no obvious homogenous nucleation was observed during this study.

## Additional Information

**How to cite this article:** Chu, B. *et al*. Influence of metal-mediated aerosol-phase oxidation on secondary organic aerosol formation from the ozonolysis and OH-oxidation of α-pinene. *Sci. Rep.* **7**, 40311; doi: 10.1038/srep40311 (2017).

**Publisher's note:** Springer Nature remains neutral with regard to jurisdictional claims in published maps and institutional affiliations.

## Supplementary Material

Supplementary Information
