# Supplementary material for: Influence of metal-mediated aerosol-phase oxidation on secondary organic aerosol formation from the ozonolysis and OH-oxidation of α-pinene
Source: Sci Rep. 2017 Jan 6;7:40311. doi: 10.1038/srep40311 (PMC5216392; doi:10.1038/srep40311)
Supplement: Supplementary Information [file srep40311-s1.doc]

**Supplementary Information**

**Influence of metal-mediated aerosol-phase oxidation on secondary organic aerosol formation from the ozonolysis and OH-oxidation of α-pinene**

*Biwu Chu ^1, 2^, John Liggio ^3^, Yongchun Liu ^1,2,4^,* *Hong He ^1,2,4,*^, Hideto Takekawa ^5^, Shao-Meng Li ^3^, Jiming Hao ^6^*

^1^ State Key Joint Laboratory of Environment Simulation and Pollution Control, Research Center for Eco-Environmental Sciences, Chinese Academy of Sciences, Beijing 100085, China

^2^ Center for Excellence in Regional Atmospheric Environment, Institute of Urban Environment, Chinese Academy of Sciences, Xiamen 361021, China

^3^ Air Quality Research Division, Environment Canada, Toronto, Ontario M3H5T4, Canada

^4^ University of Chinese Academy of Sciences, Beijing 100049, China

^5^ Toyota Central Research and Development Laboratory, Nagakute, Aichi 480-1192, Japan

^6^ State Key Joint Laboratory of Environment Simulation and Pollution Control, School of Environment, Tsinghua University, Beijing 100084, China

Correspondence and requests for materials should be addressed to H. H. ([honghe@rcees.ac.cn](mailto:honghe@rcees.ac.cn))

**Contents**

[**Experimental conditions**](#__RefHeading___Toc464320002)

[**Hygroscopic growth curves of some sulfate particles**](#__RefHeading___Toc464320003)

[**Time variations of generated gas-phase compounds from ozonolysis**](#__RefHeading___Toc464320004) [**and OH-oxidation of α-pinene**](#__RefHeading___Toc464320005)

[***f*_44_ and O/C atomic ratio as a function of *f*_43_ from ozonolysis and OH-oxidation of *α*-pinene**](#__RefHeading___Toc464320006)

[***f*_44_ of different seed aerosols**](#__RefHeading___Toc464320007)

[**One-way ANOVA statistical analysis results and means comparison with Dunn-Sidak test for SOA with different seed aerosol**](#__RefHeading___Toc464320008)

[**Possible influence of iron concentrations on their effects on SOA formation**](#__RefHeading___Toc464320009)

**Experimental conditions**

The experimental conditions of α-pinene ozonolysis experiments, including the initial concentrations of O_3_ and α-pinene and the mode diameter and concentrations (number, surface area and volume) of the seed aerosols, are listed in Table S1. Five types of seed aerosols were chosen in our experiments, including dry ferrous sulfate (FeSO_4_), dry ferric sulfate (Fe_2_(SO_4_)_3_), dry zinc sulfate (ZnSO_4_), dry ammonium sulfate (ASd), and wet ammonium sulfate (ASw). For each type of sulfate seed aerosol three experiments were carried out under similar conditions, which are numerically distinguished (i.e. 1, 2, 3 in the “Experiment No.” column) in Table S1. All experiments in Table S1 were carried out at a temperature of 23 ± 1 ℃ and 49.5 ± 1% RH.

1. Initial experimental conditions of α-pinene ozonolysis experiments

| **Experiment No.^*^** | **O_3_**  ppb | **α-pinene^**^**  ppb | **Seed aerosols** | | | | |  |
| --- | --- | --- | --- | --- | --- | --- | --- | --- |
|  |  |  | **Mode diameter**  nm | **Number**  # cm^-3^ | **Surface Area**  μm^2^ cm^-3^ | **Volume**  μm^3^ cm^-3^ | **Mass^***^**  μg m^-3^ | |
| O_3_-ASd-1 | 9.6 | 14.2 | 76 | 3900 | 85.0 | 1.3 | 2.3 | |
| O_3_-ASd-2 | 10.0 | 12.9 | 76 | 3800 | 88.0 | 1.4 | 2.5 | |
| O_3_-ASd-3 | 11.2 | 13.0 | 79 | 4000 | 93.6 | 1.5 | 2.7 | |
| O_3_-ASw-1 | 9.4 | 13.3 | 79 | 3800 | 88.9 | 1.3 | 2.3 | |
| O_3_-ASw-2 | 10.5 | 14.1 | 76 | 4000 | 85.6 | 1.2 | 2.1 | |
| O_3_-ASw-3 | 11.0 | 13.5 | 76 | 3900 | 88.4 | 1.4 | 2.5 | |
| O_3_-Fe(II)-1 | 9.9 | 13.4 | 79 | 3800 | 88.3 | 1.3 | 2.5 | |
| O_3_-Fe(II)-2 | 10.8 | 14.5 | 82 | 3800 | 99.9 | 1.6 | 3.0 | |
| O_3_-Fe(II)-3 | 11.5 | 13.8 | 76 | 3900 | 89.4 | 1.4 | 2.7 | |
| O_3_-Fe(III)-1 | 9.8 | 13.2 | 79 | 3800 | 96.0 | 1.6 | 5.0 | |
| O_3_-Fe(III)-2 | 10.4 | 12.9 | 76 | 3900 | 89.6 | 1.4 | 4.3 | |
| O_3_-Fe(III)-3 | 11.4 | 14.8 | 76 | 4000 | 93.7 | 1.4 | 4.3 | |
| O_3_-Zn-1 | 9.4 | 13.0 | 79 | 4000 | 95.6 | 1.5 | 3.0 | |
| O_3_-Zn-2 | 10.5 | 14.0 | 82 | 3800 | 98.0 | 1.6 | 3.2 | |
| O_3_-Zn-3 | 11.1 | 13.4 | 79 | 4100 | 100.3 | 1.5 | 3.0 | |

^*^ASd=dry ammonium sulfate; ASw=wet ammonium sulfate; Fe(II)=FeSO_4_; Fe(III)=Fe_2_(SO_4_)_3_; Zn=ZnSO_4_

^**^The initial α-pinene concentration is the average concentration of α-pinene between 10-20 minutes after introducing α-pinene.

^***^Assuming particle density of 1.770 g cm^-3^, 1.897 g cm^-3^, 3.097 g cm^-3^ and 1. 970 g cm^-3^ for (NH_4_)_2_SO_4_, FeSO_4_, Fe_2_(SO_4_)_3_) and ZnSO_4_, respectively.

The experimental conditions of OH-initiated oxidation of α-pinene are given in Table S2. All experiments were carried out at a temperature of 28 ± 1 ℃ and 49.5 ± 1% RH. A similar amount of ozone (about 16 ppb after 4.5 hours of reaction) was generated in these experiments. Based on the reaction rate between α-pinene and O_3_, less than 5% of the α-pinene was consumed by secondary O_3_ in the OH-oxidation experiments.

1. Initial experimental conditions of α-pinene OH-oxidation experiments

| **Experiment No.^*^** | **HONO**  ppb | **α-pinene**  ppb | **Seed aerosols** | | | | |
| --- | --- | --- | --- | --- | --- | --- | --- |
|  |  |  | **Mode diameter**  nm | **Number**  # cm^-3^ | **Surface Area**  μm^2^ cm^-3^ | **Volume**  μm^3^ cm^-3^ | **Mass****  μg m^-3^ |
| OH-Fe(II)-1 | ~9 | 11.8 | 76 | 4400 | 100 | 2.0 | 3.8 |
| OH-Fe(II)-2 | ~9 | 10.1 | 74 | 4100 | 116 | 1.6 | 3.0 |
| OH-Fe(II)-3 | ~9 | 10.0 | 71 | 4100 | 115 | 2.2 | 4.2 |
| OH-Fe(III)-1 | ~9 | 13.2 | 76 | 3600 | 82.0 | 1.3 | 4.0 |
| OH-Fe(III)-2 | ~9 | 12.4 | 71 | 3900 | 105 | 2.0 | 6.2 |
| OH-Fe(III)-3 | ~9 | 10.9 | 71 | 3800 | 106 | 2.1 | 6.5 |
| ASw | ~9 | 11.0 | 74 | 4000 | 102 | 1.7 | 3.0 |
| ASd | ~9 | 11.7 | 71 | 3900 | 100 | 1.8 | 3.2 |

^*^Fe(II)=FeSO_4_; Fe(III)=Fe_2_(SO_4_)_3_); ASw= wet ammonium sulfate; ASd=dry ammonium sulfate

**Assuming particle density of 1.897 g cm^-3^, 3.097 g cm^-3^ and 1.77 g cm^-3^ for FeSO_4_ , Fe_2_(SO_4_)_3_) and (NH_4_)_2_SO_4_, respectively.

The concentrations of FeSO_4_ seed aerosol in this in this study are about 2.5-3.0 μg m^-3^ in the ozonolysis experiments, while the concentrations of Fe_2_(SO_4_)_3_ seed aerosol are about 4.3-5.0 μg m^-3^. Considering the crystal water content in FeSO_4_·7H_2_O and the hygroscopic growth of the two seed aerosols (Figure S1), the iron concentrations were estimated to be 0.35-0.42 μg m^-3^ and 0.97-1.13 μg m^-3^ in experiments with FeSO_4_ and Fe_2_(SO_4_)_3_, respectively. As for the concentrations of iron in the particles, the iron contributed to about 14% and 23% of the particle mass at the beginning of experiments with FeSO_4_ and Fe_2_(SO_4_)_3_, respectively. At the end of the experiments, with the generated SOA counted in, iron contributed to 5% and 10% of the particle mass at the end of experiments with FeSO_4_ and Fe_2_(SO_4_)_3_ , respectively. The concentrations of iron in OH-oxidation experiments were calculated similarly and are listed in Table S3.

1. Iron concentrations in the experiments

| **Oxidation system** | **Seed aerosol** | **Seed aerosol concentration**  **(μg m^-3^)** | **Iron concentration in the chamber**  **(μg m^-3^)** | **Iron concentration in the particle phase** | |
| --- | --- | --- | --- | --- | --- |
|  |  |  |  | **Beginning of experiment (mg/g-particle)** | **End of experiment**  **(mg/g-particle)** |
| Ozonolysis | FeSO_4_ | 2.5-3.0 | 0.35-0.42 | 140 | 44-51 |
|  | Fe_2_(SO_4_)_3_ | 4.3-5.0 | 0.97-1.13 | 225 | 97-105 |
| OH-oxidation | FeSO_4_ | 3.0-4.2 | 0.42-0.59 | 140 | 58-70 |
|  | Fe_2_(SO_4_)_3_ | 4.0-6.5 | 0.90-1.46 | 225 | 94-121 |

**Hygroscopic growth curves of some sulfate particles**


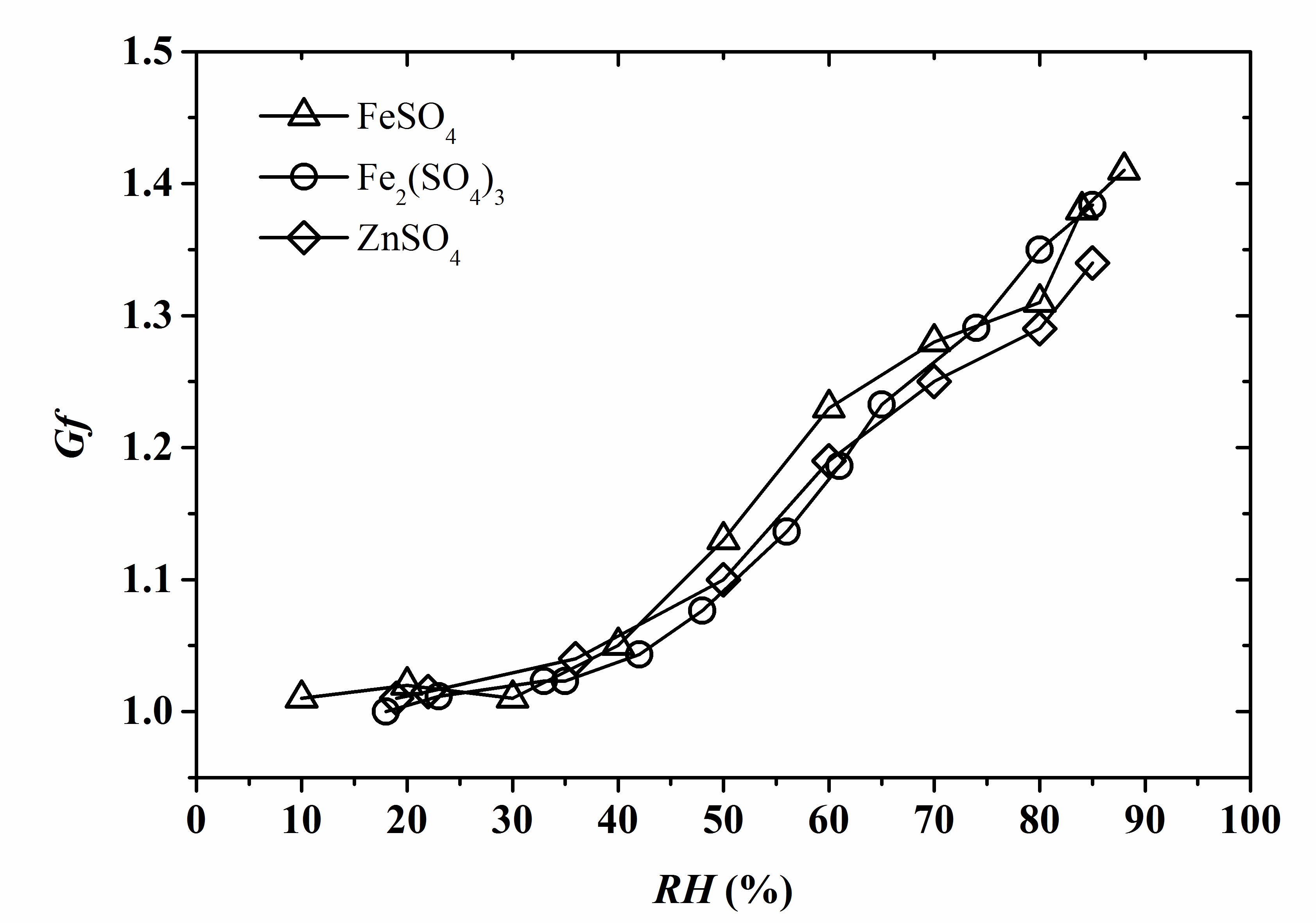


**Figure S1. Hygroscopic growth curves of FeSO_4_, Fe_2_(SO_4_)_3_ and ZnSO_4_ particles.** The hygroscopic growth factor (*Gf*) was measured using particles with initial dry diameter (*d*_p0_) of 100 nm.

**Time variations of generated gas-phase compounds from ozonolysis and OH oxidation of α-pinene**

**Figure S2. Variations of *α*-pinene and generated gas phase compounds as a function of reaction time from ozonolysis of *α*-pinene with different sulfate seed aerosols.** The amounts of α-pinene consumed after 4.5 hours of reaction are shown in the inset of (a), while the abundances of acetone, pinonaldehyde and pinic acid relative to the consumed α-pinene are shown in the inset of (b), (c) and (d), respectively. For pinonaldehyde and gas-phase pinic acid, we simply compared the normalized signal strength of representative fragments since we did not purchase a standard gas for them. To remove some high frequency noise, the traces were smoothed with a time period of 10 minutes. Five types of seed aerosols were introduced in these experiments, including dry ferrous sulfate (Fe(II)), dry ferric sulfate (Fe(III)), dry zinc sulfate (Zn), dry ammonium sulfate (ASd), and wet ammonium sulfate (ASw).


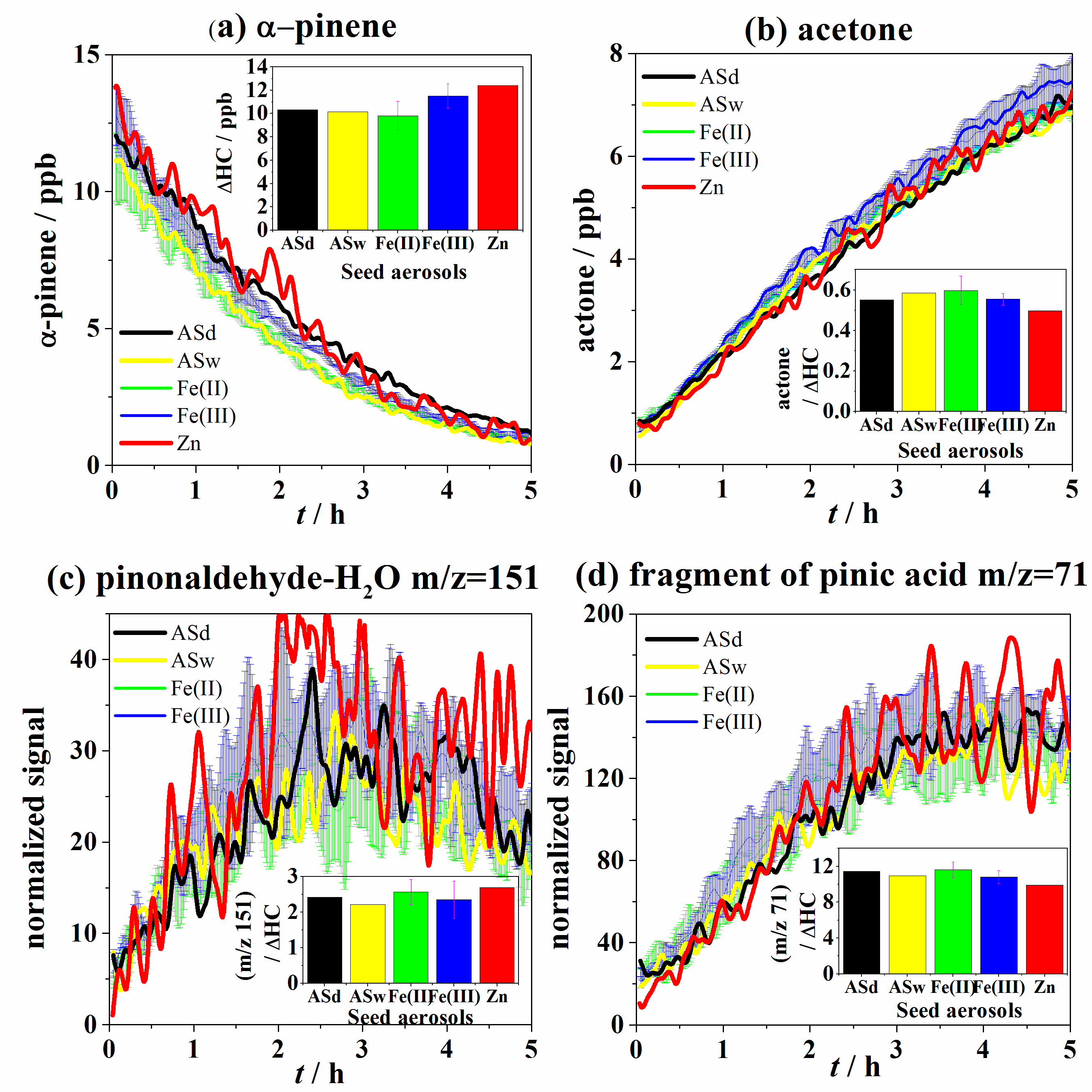


**Figure S3.** **Variations of *α*-pinene and generated gas phase compounds as a function of reaction time from OH-oxidation of *α*-pinene with different sulfate seed aerosols.** The amounts of α-pinene consumed after 4.5 hours of reaction are shown in the inset of (a), while the abundances of acetone, pinonaldehyde and pinic acid relative to the consumed α-pinene are shown in the inset of (b), (c) and (d), respectively. For pinonaldehyde and gas-phase pinic acid, we simply compared the normalized signal strength of representative fragments since we did not purchase a standard gas for them. To remove some high frequency noise, the traces were smoothed with a time period of 10 minutes. Five types of seed aerosols were introduced in these experiments, including dry ferrous sulfate (Fe(II)), dry ferric sulfate (Fe(III)), dry zinc sulfate (Zn), dry ammonium sulfate (ASd), and wet ammonium sulfate (ASw).

***f*_44_ and O/C atomic ratio as a function of *f*_43_ from ozonolysis and OH-oxidation of *α*-pinene**


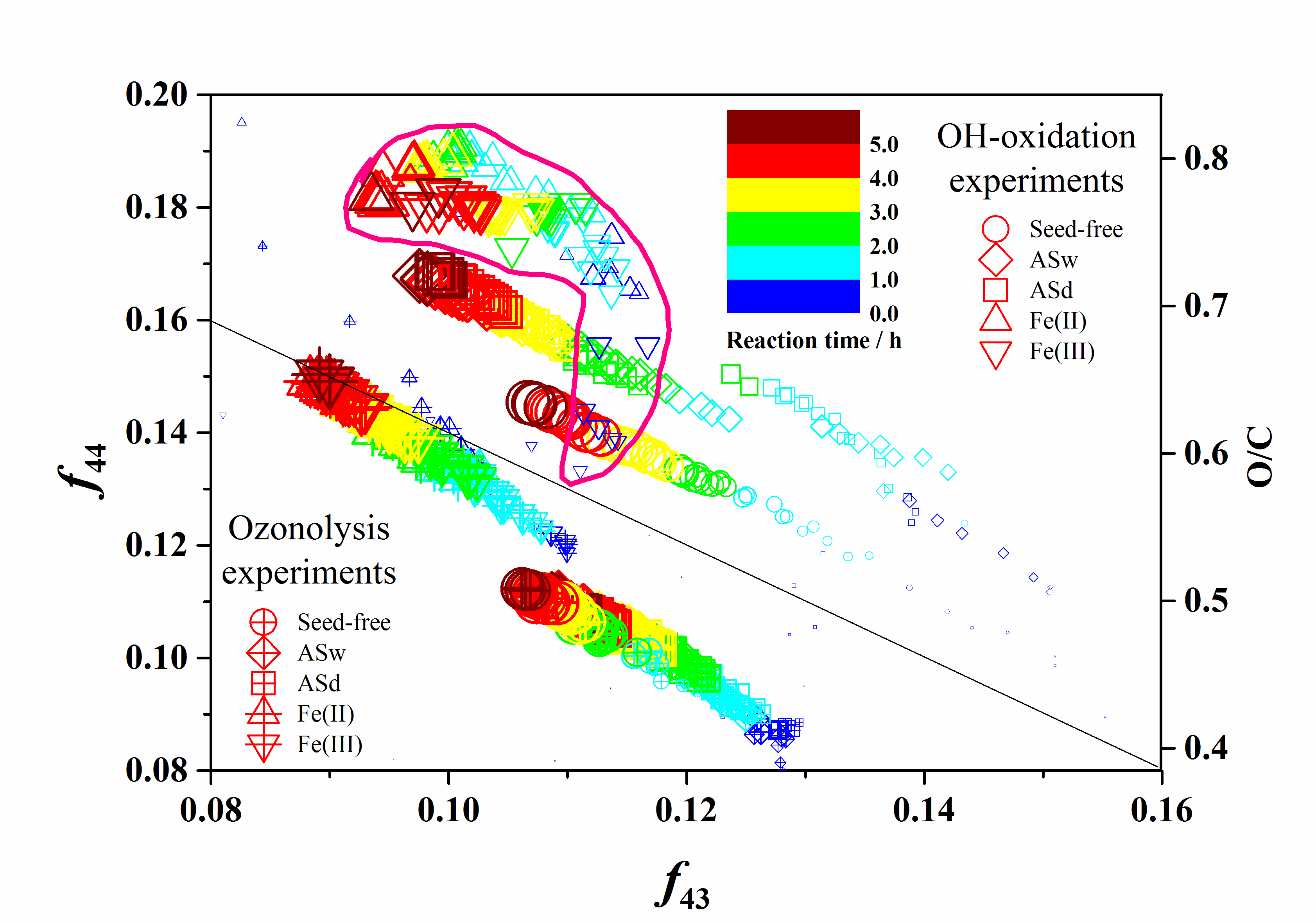


**Figure S4.** ***f*_44_ and the estimated O/C atomic ratio as a function of *f*_43_ from ozonolysis and OH-oxidation of *α*-pinene.** The color and the size of symbols indicate the reaction time and the SOA mass concentration, respectively.

***f*_44_ of different seed aerosols**


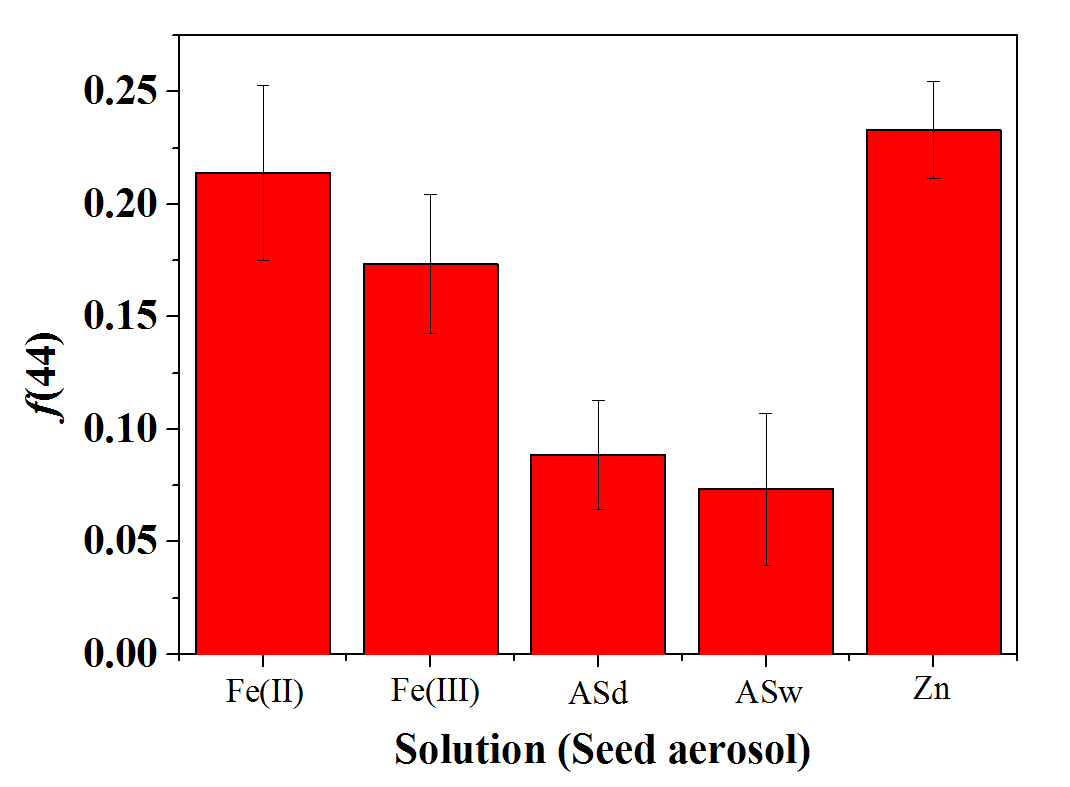


Figure S5. *f*(44) values for metal solutions and ammonium sulfate solutions from AMS

**One-way ANOVA statistical analysis results and means comparison with Dunn-Sidak test for SOA with different seed aerosol**

Table S4. One-way ANOVA statistical analysis results and means comparison with Dunn-Sidak test for SOA yields in α-pinene+O_3_ with different seed aerosols (significance level: 0.05):

(a) SOA yields: F=37.66941, P=5.31184E-6

| **Seed aerosols** | **Liquid (NH_4_)_2_SO_4_** | **FeSO_4_** | **Fe_2_(SO_4_)_3_** | **ZnSO_4_** |
| --- | --- | --- | --- | --- |
| **Dry** **(NH_4_)_2_SO_4_** | P= 0.68543  Not significant | P= 3.8189E-5  Significant | P= 2.65647E-5  Significant | P= 0.1219  Not significant |
| **Liquid (NH_4_)_2_SO_4_** |  | P= 2.53374E-4  Significant | P= 1.65349E-4  Significant | P= 0.93223  Not significant |
| **FeSO_4_** |  |  | P= 1  Not significant | P=0.00122  Significant |
| **Fe_2_(SO_4_)_3_** |  |  |  | P= 7.50188E-4  Significant |

(b) SOA mass: F= 3.74915, P= 0.04098

| **Seed aerosols** | **Liquid (NH_4_)_2_SO_4_** | **FeSO_4_** | **Fe_2_(SO_4_)_3_** | **ZnSO_4_** |
| --- | --- | --- | --- | --- |
| **Dry (NH_4_)_2_SO_4_** | P= 1  Not significant | P= 0.30514  Not significant | P= 0.15002  Not significant | P= 1  Not significant |
| **Liquid (NH_4_)_2_SO_4_** |  | P= 0.44846  Not significant | P= 0.23295  Not significant | P= 1  Not significant |
| **FeSO_4_** |  |  | P= 0.99997  Not significant | P= 0.44149  Not significant |
| **Fe_2_(SO_4_)_3_** |  |  |  | P= 0.22864  Not significant |

(c) Oxidation level: F=388.13535, P= 6.40724E-11

| **Seed aerosols** | **Liquid (NH_4_)_2_SO_4_** | **FeSO_4_** | **Fe_2_(SO_4_)_3_** | **ZnSO_4_** |
| --- | --- | --- | --- | --- |
| **Dry (NH_4_)_2_SO_4_** | P= 0.96086  Not significant | P= 6.73704E-10  Significant | P= 9.72137E-10  Significant | P= 7.91219E-5  Significant |
| **Liquid (NH_4_)_2_SO_4_** |  | P= 1.01328E-9  Significant | P= 1.48478E-9  Significant | P= 2.86038E-4  Significant |
| **FeSO_4_** |  |  | P= 0.9803  Not significant | P=2.08093E-8  Significant |
| **Fe_2_(SO_4_)_3_** |  |  |  | P= 3.49158E-8  Significant |

Table S5. One-way ANOVA statistical analysis results and means comparison with Dunn-Sidak test for SOA yields in OH-oxidation of α-pinene with different seed aerosols (significance level: 0.05):

(a) SOA yields: F=56.64704, P=8.92309E-4

| **Seed aerosols** | **Liquid (NH_4_)_2_SO_4_** | **ZnSO_4_** | **FeSO_4_** | **Fe_2_(SO_4_)_3_** |
| --- | --- | --- | --- | --- |
| **Dry (NH_4_)_2_SO_4_** | P= 0.99998  Not significant | P= 0.90823  Not significant | P= 0.00805  Significant | P= 0.01151  Significant |
| **Liquid (NH_4_)_2_SO_4_** |  | P= 0.72641  Not significant | P= 0.01037  Significant | P= 0.01518  Significant |
| **ZnSO_4_** |  |  | P= 0.00399  Significant | P=0.00539  Significant |
| **FeSO_4_** |  |  |  | P= 0.97669  Not significant |

(b) SOA mass: F= 20.59729, P= 0.00623

| **Seed aerosols** | **Liquid (NH_4_)_2_SO_4_** | **ZnSO_4_** | **FeSO_4_** | **Fe_2_(SO_4_)_3_** |
| --- | --- | --- | --- | --- |
| **Dry (NH_4_)_2_SO_4_** | P= 1  Not significant | P= 0.99998  Not significant | P= 0.04068  Significant | P= 0.08128  Not significant |
| **Liquid (NH_4_)_2_SO_4_** |  | P= 0.9993  Not significant | P= 0.04854  Significant | P= 0.09957  Not significant |
| **ZnSO_4_** |  |  | P= 0.02885  Significant | P= 0.0548  Not significant |
| **FeSO_4_** |  |  |  | P= 0.90278  Not significant |

(c) Oxidation level: F=1.00495, P= 0.49815

| **Seed aerosols** | **Liquid (NH_4_)_2_SO_4_** | **ZnSO_4_** | **FeSO_4_** | **Fe_2_(SO_4_)_3_** |
| --- | --- | --- | --- | --- |
| **Dry (NH_4_)_2_SO_4_** | P= 1  Not significant | P= 0.99964  Not significant | P= 0.89971  Not significant | P= 0.97459  Not significant |
| **Liquid (NH_4_)_2_SO_4_** |  | P= 0.99937  Not significant | P= 0.88196  Not significant | P= 0.9665  Not significant |
| **ZnSO_4_** |  |  | P= 0.99934  Not significant | P=1  Not significant |
| **FeSO_4_** |  |  |  | P= 0.99998  Not significant |

**Possible influence of iron concentrations on their effects on SOA formation**

The mass concentrations of iron (0.35-1.46 μg m^-3^) in the chamber in this study were comparable to the total concentration of iron in the atmosphere in polluted cities. For example, the iron concentration in PM_2.5_ in Beijing were reported in the range of 0.76-1.32 μg m^-3^ ^ref.^[^1^](#_ENREF_1), 0.43-1.44 μg m^-3^ ^ref.^[^2^](#_ENREF_2), 0.5-0.9 μg m^-3^ ^ref.^[^3^](#_ENREF_3) and 0.73-2.05 μg m^-3^ ^ref.^[^4^](#_ENREF_4) in different studies. However, the seed aerosols used in this study were all sulfate, and the water soluble iron concentrations would be higher than that under normal ambient conditions. Besides, as the reviewer pointed out, the concentrations of iron in the aerosol-phase were much higher than the ambient conditions since the particles in the chamber had far less components than the ambient fine particles. Iron is expected to influence SOA in the cyclic oxidation-reduction continuously according to the proposed mechanism. In this study, the SOA mass and the oxidation level became stable in only 1-2 hours, indicating a fast aerosol-phase oxidation. We tried to lower the concentrations of iron in the experiments, and found less significant impact of seed aerosols on SOA formation, which was consistent with what we reported in our previous study[^5^](#_ENREF_5). Lower concentration of iron in the ambient atmosphere is likely to cause less significant decrease of SOA mass. However, with low concentrations of seed aerosols, new particles were also generated in the oxidation system, causing some generated SOA were not partitioned to the surface of iron sulfate seed aerosols. This could also be the reason that lower concentrations of iron decreased SOA mass less significantly.

The influence of the high concentration of iron in the aerosol phase is not likely to cause an overestimation for the effects of FeSO_4_ seed aerosols on SOA formation according to some previous experimental data for m-xylene photooxidation[^6^](#_ENREF_6). In Fig. S6, we compared the decreasing amount of SOA with the presence of FeSO_4_ seed aerosols under two situations. One is that only FeSO_4_ seed aerosols were added while in the other situation, FeSO_4_ were mixed with (NH_4_)_2_SO_4_ (using mixed solutions of FeSO_4_ and (NH_4_)_2_SO_4_ with mass concentration ratios of 1:5). The mass concentrations of FeSO_4_ seed aerosols in the chamber were similar under the two conditions, while iron concentration in the aerosol-phase were much lower in the later situation since 4 times higher concentration of (NH_4_)_2_SO_4_ were added together with FeSO_4_. Since SOA were reduced more by the presence of FeSO_4_ with the co-existence of (NH_4_)_2_SO_4_ than that by FeSO_4_ alone, we conjecture that the high concentration of iron in the aerosol phase is not likely to result in to cause an overestimation, but likely to cause an underestimation for the effects of FeSO_4_ seed aerosols on SOA formation, as long as the concentration of total iron is the same in the chamber.


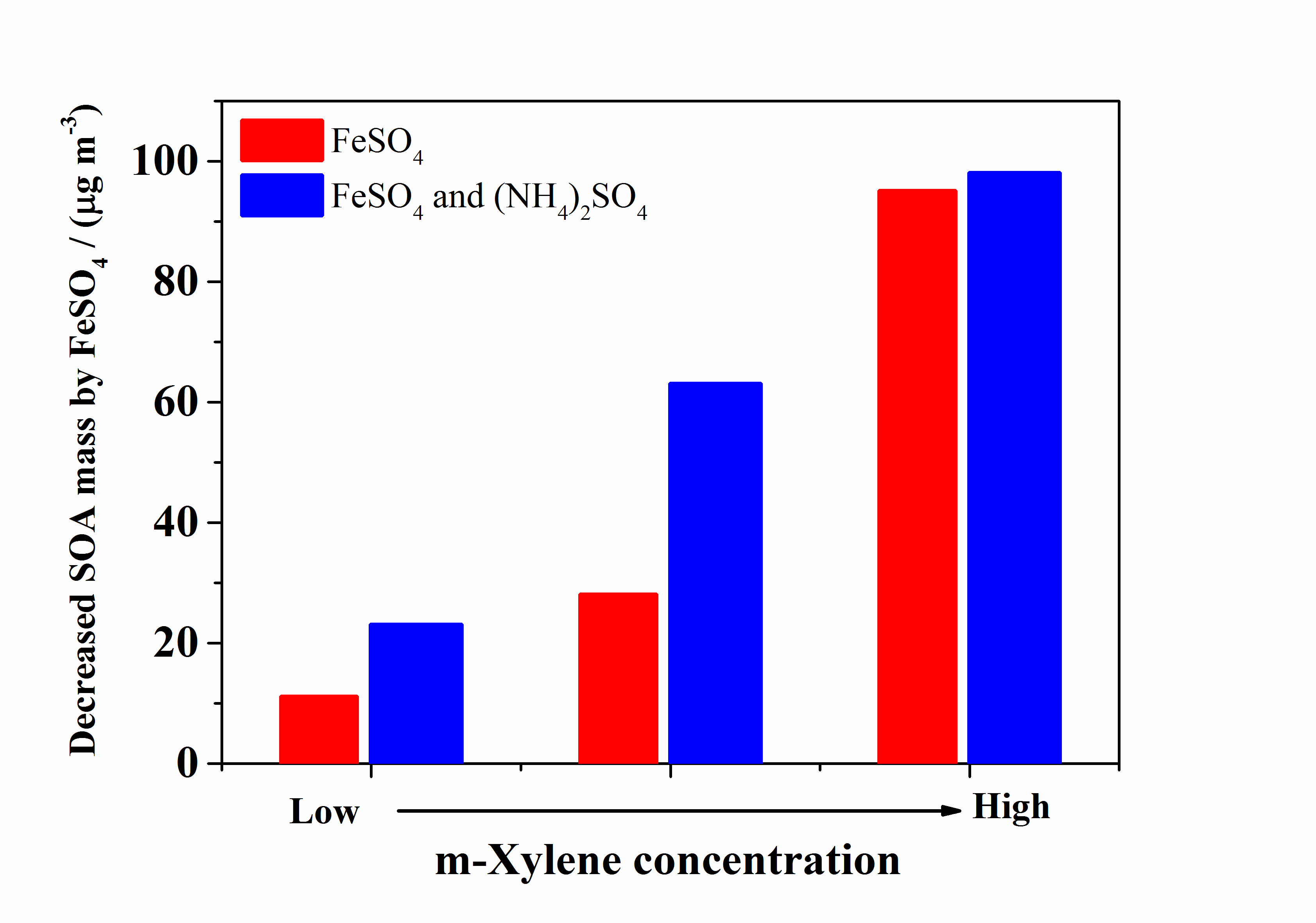


**Figure S6.** Decreasing effect of FeSO_4_ seed aerosols on SOA formation in the presence or absence of (NH_4_)_2_SO_4_ seed aerosols.

Table S6. Comparison of experimental results in with previous studies for effects of iron sulfate seed aerosols on SOA formation from α-pinene oxidation

| **studies** | **1 (ref** [**^5^**](#_ENREF_5)**)** | **2 (ref** [**^7^**](#_ENREF_7)**)** | | **This Study** | |
| --- | --- | --- | --- | --- | --- |
| **Oxidation system** | α-pinene + NOx+UV | α-pinene + NOx+UV | α-pinene + HONO+UV | α-pinene + O_3_ | α-pinene + HONO+UV |
| **VOCs Conc.** | 0.15-0.3ppm | 0.2-0.3ppm | ~10 ppb | ~10ppb | ~10ppb |
| **Iron type** | FeSO_4_ | FeSO_4_, (NH_4_)_2_SO_4_ | FeSO_4_, (NH_4_)_2_SO_4_ | FeSO_4_, Fe_2_(SO_4_)_3_, ZnSO4, (NH_4_)_2_SO_4_ | FeSO_4_, Fe_2_(SO_4_)_3_, ZnSO4, (NH_4_)_2_SO_4_ |
| **Iron Conc.** | 0.2-2.0μg m^-3^ | 1.7μg m^-3^ | 0.3μg m^-3^ | 0.3-0.4μg m^-3^ | 0.4-0.6μg m^-3^ |
| **Seed size distribution** | Polydisperse  (10-500nm) | Polydisperse  (10-500nm) | Polydisperse  (10-500nm) | Monodisperse  (70nm) | Monodisperse  (70nm) |
| **SOA loading** | 40-260μg m^-3^ | 50-320μg m^-3^ | 4-14μg m^-3^ | 4-8μg m^-3^ | 3-13μg m^-3^ |
| **New particle formation** | Uncontrolled | Uncontrolled | Uncontrolled | Controlled | Controlled |
| **SOA decrease percentage** | 8-58%  (8-34% with 0.5μg m^-3^ iron) | 54-55% | ~65% | Fe(II) or Fe(III):  ~20% | Fe(II) or Fe(III):  ~60% |
| **O/C increase percentage** | NA | NA | NA | Fe(II) or Fe(III):  +5% | Fe(II) or Fe(III):  +32% |

**References**

1 He, K. B. *et al.* The characteristics of PM_2.5_ in Beijing, China. *Atmos. Environ.* **35**, 4959-4970 (2001).

2 Song, S. *et al.* Chemical characteristics of size-resolved PM_2.5_ at a roadside environment in Beijing, China. *Environ. Pollut.* **161**, 215-221 (2012).

3 Tian, S. L., Pan, Y. P., Liu, Z. R., Wen, T. X. & Wang, Y. S. Size-resolved aerosol chemical analysis of extreme haze pollution events during early 2013 in urban Beijing, China. *J. Hazard. Mater.* **279**, 452-460 (2014).

4 Zhao, P. S. *et al.* Characteristics of concentrations and chemical compositions for PM2.5 in the region of Beijing, Tianjin, and Hebei, China. *Atmos. Chem. Phys.* **13**, 4631-4644 (2013).

5 Chu, B. *et al.* The remarkable effect of FeSO4 seed aerosols on secondary organic aerosol formation from photooxidation of α-pinene/NOx and toluene/NOx. *Atmos. Environ.* **55**, 26-34 (2012).

6 Chu, B. *et al.* Effects of Inorganic Seeds on Secondary Organic Aerosol (SOA) Formation, *Atmospheric Aerosols - Regional Characteristics - Chemistry and Physics.* (ed Hayder Abdul-Razzak) ( InTech, 2012).

7 Chu, B. *et al.* Decreasing effect and mechanism of FeSO_4_ seed particles on secondary organic aerosol in α-pinene photooxidation. *Environ. Pollut.* **193**, 88-93 (2014).
